# High‐throughput droplet‐based microfluidics for directed evolution of enzymes

**DOI:** 10.1002/elps.201900222

**Published:** 2019-08-29

**Authors:** Flora W. Y. Chiu, Stavros Stavrakis

**Affiliations:** ^1^ Institute for Chemical and Bioengineering ETH Zürich Zürich Switzerland

**Keywords:** directed evolution, droplet‐based microfluidics, high throughput screening, protein engineering, single‐cell

## Abstract

Natural enzymes have evolved over millions of years to allow for their effective operation within specific environments. However, it is significant to note that despite their wide structural and chemical diversity, relatively few natural enzymes have been successfully applied to industrial processes. To address this limitation, directed evolution (DE) (a method that mimics the process of natural selection to evolve proteins toward a user‐defined goal) coupled with droplet‐based microfluidics allows the detailed analysis of millions of enzyme variants on ultra‐short timescales, and thus the design of novel enzymes with bespoke properties. In this review, we aim at presenting the development of DE over the last years and highlighting the most important advancements in droplet‐based microfluidics, made in this context towards the high‐throughput demands of enzyme optimization. Specifically, an overview of the range of microfluidic unit operations available for the construction of DE platforms is provided, focusing on their suitability and benefits for cell‐based assays, as in the case of directed evolution experimentations.

**Abbreviations**
DE: directed evolution
FACS: fluorescence‐activated cell sorting
FADS: Fluorescence‐activated droplet sorting
IVC: in vitro compartmentalization
MTP: microtitre plate
PFPE: perfluoropolyether
w/o: water‐in‐oil
w/o/w: water‐in‐oil‐in‐water

## 1 Introduction

Living organisms often take millions of years to evolve enzymes capable of metabolizing naturally occurring substances. Enzymes are exquisitely versatile and proficient catalysts. Optimized by evolution over time, they can initiate, accelerate, and control a diversity of reactions within living systems, whilst ensuring high substrate specificity as well as extraordinary selectivity. Despite their wide structural and chemical diversity, relatively few natural enzymes have been successfully applied to the processing of non‐living systems in industrial settings 1. This is due, in large part, to the fact that prediction, optimization, and tuning of enzyme properties is ultimately defined by a quantitative understanding of their chemical structure, interaction with substrate molecules, and their temporal dynamics. Furthermore the costs associated with their in vitro production, or their adoption in commercial‐scale processes have been limiting factors for improving industrial enzyme catalysis 2. In recent years, an increasing number of methods have been successfully used to augment our understanding of such biological systems and assist in the identification of enzymes with tailored properties. Of these, the practical evolution of enzymes concerning the conversion of non‐natural, anthropogenic compounds 3 relies largely on directed, or in vitro, evolution; a protein engineering approach that enables the generation of bespoke enzymes 4, 5. This method involves simulating natural evolution in the lab, where mutant libraries of target enzyme are created and then screened for required properties. Directed evolution (DE) has shown success in improving properties such as enzyme activity, stability, and substrate selectivity, and has begun to transform the field of protein engineering 6.

**Figure 1:**
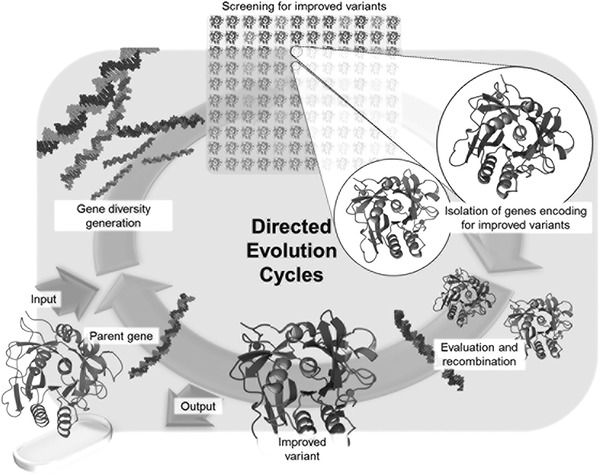
Schematic representation of a directed evolution campaign. The campaign comprises iterative rounds in which a parent gene, coding for an enzyme of interest, is subjected to random mutagenesis (or rational design) to yield a mutant library. Enzyme variants are subsequently expressed and screened for a desired property, such as activity. Improved variants are isolated, evaluated and then used to parent the next round of the evolution cycle. Reprinted and reproduced from 4, with the permission of SciELO Publishing.

Directed evolution coheres with Darwinian evolution in two ways. First, a genetically diverse “population” (or gene library) is created and translated into a corresponding library of gene products (encoded enzymes). This is then followed by a selection process aimed at isolating variants with the most‐desired characteristics (the “fittest”) 7. In theory, such an approach requires no a priori knowledge of the protein structure. Moreover, the process is iterative, with the gene(s) encoding improved performance being identified and used to parent the next round of evolution (Fig. 1) 4. The most straightforward way to generate a library having substantial genetic diversity is via random mutagenesis, as can be accomplished in the laboratory using various molecular biology techniques such as error‐prone PCR 8, saturation mutagenesis 9, and DNA‐shuffling 10, 11. That said, creating a library that covers the entirety of mutational space is impossible in practice. For example, complete randomization of a mere decapeptide would require 10^13^ unique combinations of amino acids (20 possible amino acid building blocks per position; 1 × 20^10^ ≈ 1 × 10^13^), which exceeds the achievable library size using any common protein library creation method 5. Additionally, even if screening at a rate of one million variants per hour, one year of continuous screening would cover less than 0.1% of variants within the library. Given that enzymes typically contain between 180 and 600 amino acids, protein engineers are motivated to devise methods that can create libraries of accessible (and manageable) size, whilst at the same time, maximizing the likelihood of identifying improved variants within a library 5, 12. For example, when structural information regarding a target enzyme is available, rational design and DE methods can be combined to create smaller more focused libraries. As such, genetic variations are typically only introduced at functional sites, focusing on a few amino acid residues. So far, many successful attempts to modify enzymatic enatioselectivity and substrate specificity have been reported, based on such approach 13, 14.

**Figure 2:**
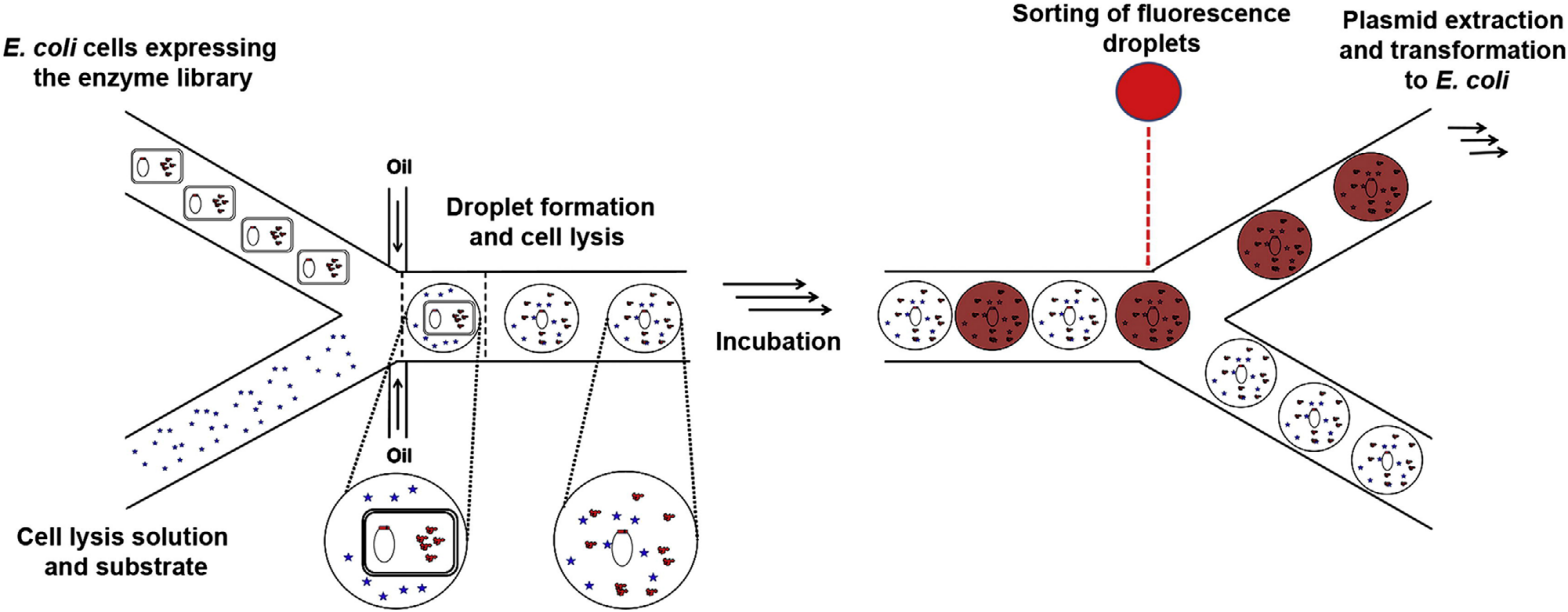
Generic workflow of microfluidic sorting. Single cells expressing a library of recombinant enzymes together with cell lysis solution and substrate are encapsulated within pL‐volume droplets and their contents are mixed. Subsequently, droplets are incubated and then sorted according to the fluorescence signal. Reprinted and reproduced from 16, with the permission of Elsevier Publishing.

Although the realization of improved mutants via DE strategies has been reported, variants with even higher activities are in most cases anticipated, since the number of variants that have been screened is miniscule when compared to the scope of available gene sequence space. Indeed, the structural characterization of enzymes and mutant library design together represents only the first step en route to the discovery of novel enzymes. Given the vast libraries of mutants that can in principle result from all kinds of engineering activities, tools and technologies that allow for the creation of high‐throughput screening platforms are equally, if not more, valuable. Droplet‐based microfluidic technologies have been shown to hold enormous potential for high‐throughput screening applications 15. Recently, high‐throughput platforms for screening large populations of biological samples for DE of enzymes have been reported in the literature (See Section 3 and 4). Specifically, pL‐volume droplets (each containing a single cell) can be formed and sorted at kHz frequencies, which in turn yields a population of droplets containing the most active enzymes 16 (Fig. 2). The sorting strategy involves assessing enzymatic efficiency through measurement of the time integrated fluorescence signal originating from individual droplets.

## 2 Conventional methods for enzyme selection and screening

The scale of DE experiments is generally limited by access to high‐throughput screening methods, since the number of variants that can be realistically screened are orders of magnitude smaller than the typical library size. Put simply, the development of assays that allow rapid and cost‐effective selection of desirable mutants is demanded. High‐throughput screening is a poorly defined term, but is generally understood to describe platforms and methods able to perform between 10^4^ and 10^5^ tests per day 17. Based on this definition, methods able to screen more than 10^6^ compounds per day can be considered as ultra‐high‐throughput. Although assays for specific enzyme families are normally tailored according to the reactions involved, a common principle is followed. First, a reliable enzyme production system is required. Based on their high transformation efficiencies and well‐established genetic manipulation tools, *Escherichia coli* (*E. coli*), *Bacillus subtilis* (*B. subtilis*) and *Saccharomyces cerevisiae* (*S. cerevisiae*) are the most widely used host organisms 18. The most important aspect in this regard is that enzyme production should be sufficiently high to allow the facile detection of activity and the expression of protein is homogeneous across host population. Second, the output of the assay (e.g., fluorescence emission or absorbance) should be directly associated with the property of interest (e.g., enzyme activity or thermostability). Readout of the assay will also define available instruments for measurements and relevant detection limits. Lastly, the substrate should be identical with, or at least similar enough to the target substrate. In many cases, the use of “real” substrates becomes impossible due to limited availability or economic reasons, so instead “model” substrates are used. This may seem to be more convenient at the start of an investigation but might lead to optimization of enzyme towards the ‘’model” substrate and not necessarily the “real” substrate.

**Figure 3:**
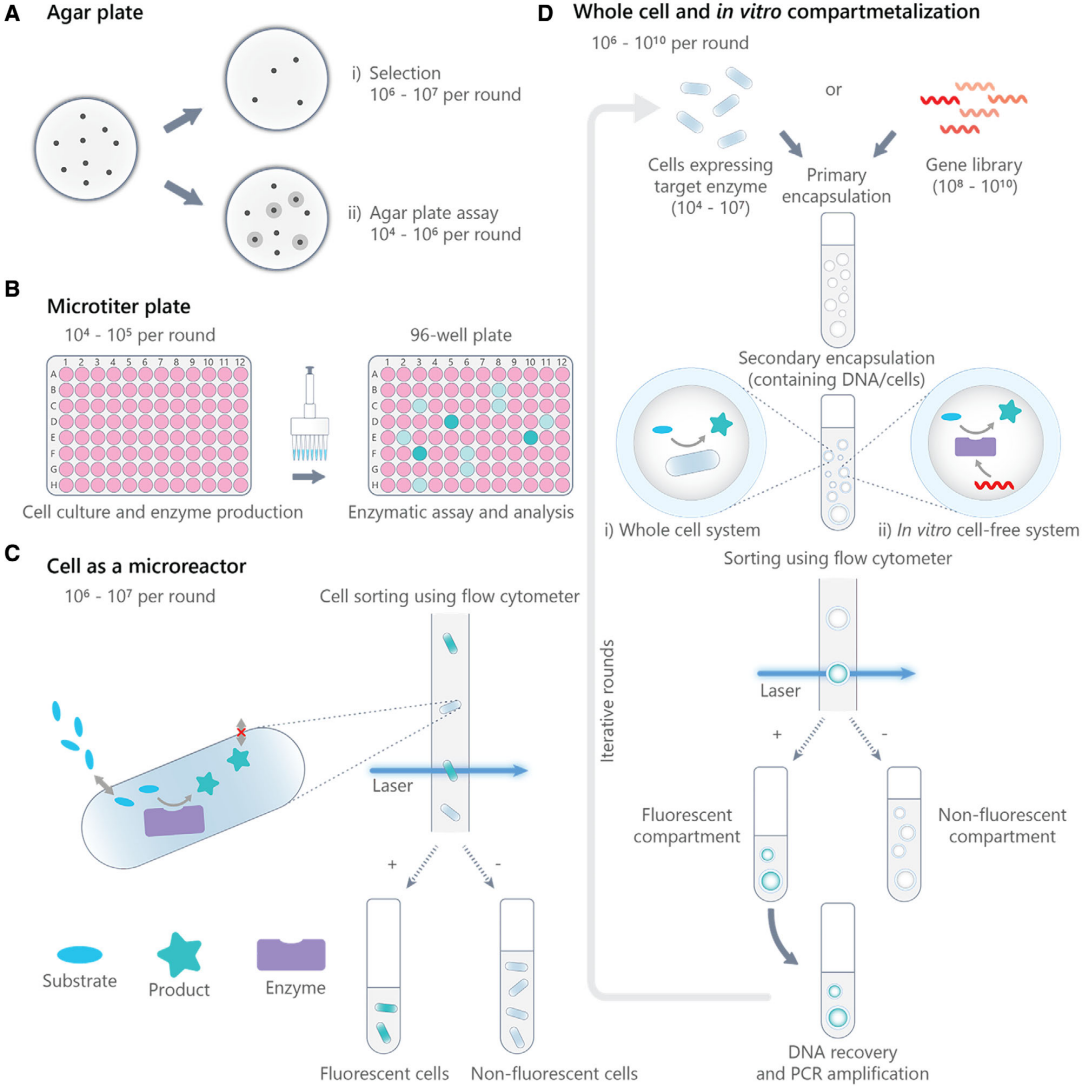
Conventional library screening methods for the directed evolution of enzymes. (A) (i) Selection screening and (ii) agar‐plate assays. (B) Microtiter‐plate (MTP) screening. (C) Cell‐based compartmentalization coupled with fluorescence‐activated cell sorting (FACS). This approach relies on either the substrate being able to freely diffuse across the cell membrane to reach cytoplasmically expressed enzyme, or enzyme being displayed on the cell surface to react with substrate. (D) (i) Whole‐cell and (ii) in vitro compartmentalization (IVC) coupled with FACS, where sorting is made possible by the production of w/o/w double emulsions. Reprinted and reproduced from 4, with the permission of SciELO Publishing.

Screening methods that rely on the direct link between cell growth and improved enzyme functionality are collectively known as selection screenings or selection assays (Fig. 3Ai). Cells are first transformed with a library of mutant genes (such that each cell expresses one distinct protein variant). The transformed cells are then grown in a selective medium that lacks essential nutrients or contains toxic compounds 19. As such, only variants exhibiting favorable activity survive. Selection screenings are compatible with large libraries (up to 10^7^ per round of evolution) and inexpensive. However, such a method is only possible when enzyme activity offers a growth advantage in host cells, significantly limiting its application. Next in the complexity scale and throughput are agar plate assays, where substrate conversion creates a visual signal (such as a color or fluorescence variation) around active colonies within the agar medium 20. Agar plate assays are easy to perform but less suitable for quantifying the catalytic activity of individual enzyme variants within a library. Additionally, analytical throughput is relatively low (∼10^4^ per round of evolution), making agar plate assays more widely used as a pre‐screening tool (Fig. 3Aii).

Microtitre plate (MTP) screening is still the most commonly used format for library screening. It represents a miniature analogue of the cuvette system, where the balance between assay complexity and throughput is well‐distributed. In particular, the possibility to couple MTPs with many analytical tools and standardized equipment for detection has made it exceptionally practical. A standard plate reader can readily perform UV‐Vis absorbance and fluorescence detection of samples in a 96‐well plate. Analysis of the contents of each microwell using LC/MS or GC/MS can also be realized 21. Accordingly, quantitative activity measurements on each variant can be obtained, providing a more reliable dataset for library evaluation when compared to selection assays. In a typical assay, transformed cells are first inoculated and grown in a 96‐well plate (the master plate). Cells are then transferred to a second plate (the expression plate) in which enzymes are produced and extracted (by cell lysis). Finally, the enzymatic reaction is initiated by the addition of substrate (Fig. 3B). With the recent advancements in liquid‐handling technologies and robotics, an increase in MTP well‐density from 96‐ (100–200 µL per well) to 384‐ (30–100 µL per well), and even 1536‐well (2.5–10 µL per well) has been reported 22. Moreover, by replacing wells with microcapillaries of volumes as small as ∼200 nL, number of samples accommodated per standard‐sized plate can even be further increased to 100 000 (called the Giga Matrix 23). Although such small volumes have imposed other challenges associated with capillarity and uncontrollable evaporation, hence sample recovery.

Further miniaturization of reaction systems has led to increased interest in using single host cells as reaction compartments, with the activity of each enzyme variant being assessed by fluorescence‐activated cell sorting (FACS). FACS is a powerful technique that allows screening and sorting of cells at rates of up to 400 000 cells per second. This means that, in principle, the screening of 1 million variants can be accomplished in less than 2 min. In addition, the assay volume in this case has effectively decreased from a few microliters (in a MTP well) to approximately the volume of single bacterial cell, which is typically a few femtoliters. Yet, unlike MTP‐based assays, where each well acts as a physical boundary to enclose the reaction mixture, the use of FACS requires the physical linkage between genotype (the enzyme‐encoding gene) and phenotype (a functional trait, e.g., catalytic activity) to be confined at a single‐cell level. This ensures the possibility to trace back and identify beneficial gene mutations accordingly. In the context of an enzymatic reaction, the assay is restricted to two main scenarios. In the first case, the enzyme produced stays within the cytoplasm of the cell. The substrate should then have the ability to diffuse across the cell membrane for reaction to occur, whilst the fluorescent product accumulates and stays trapped inside cells. In the second scenario, the enzyme is displayed at the surface of the cell to facilitate reaction with substrate and at the same time, the fluorescent product is also captured on the cell surface (Fig. 3C). By exploiting the selective entrapment of fluorescent reaction products within *E. coli* cells, Aharoni et al. reported the screening of > 10^6^ glycosyltransferase variants from an error‐prone PCR library using FACS 24. On the other hand, the surface display technique is more frequently used to improve binding affinity and stability of a protein, e.g., engineering of antibodies, and only in a fewer case is applied in screening of enzyme mutants. One example is the optimization of enantioselectivity of the *Pseudomonas aeruginosa* esterase EstA by displaying enzyme variants on the surface of *E. coli*. Esterase activity results in the release of a phenolic compound which in turn is captured on cell surface via peroxidase catalyzed radical formation 25. Apart from surface display on bacterial cells, mammalian cell display and yeast cell display have also been described 26. Complemented by FACS, overall screening capacity and assay throughput have been dramatically increased when using these strategies. The major drawbacks herein are perhaps the need to design and synthesize appropriate fluorescent substrates, which might not always be straightforward, and the fact that a majority of proteins simply cannot be displayed on cells.

To address the product accumulation challenge, alternative methods for compartmentalization have to be devised to ensure genotype‐phenotype linkage. To this end, emulsion‐based technologies have emerged as potential tools, where the evolved enzymes, substrate as well as the product are all encapsulated in water‐in‐oil (w/o) microdroplets 27, 28. The idea of creating man‐made compartments (as opposed to cellular compartmentalization) by forming an emulsion was first conceptualized by Tawfik and Griffiths in 1998, when they also termed the technique in vitro (i.e., cell‐free) compartmentalization (IVC) (Fig. 3Di) 29, 30. In their first report on IVC, they demonstrated the possibility to perform transcription and translation of single DNA‐methyltransferase genes within aqueous compartments of a water‐in‐oil emulsion, resulting in the production of active enzymes. Upon conversion of substrate, product generated remained linked to corresponding genes and trapped within droplets. Moreover, by applying an appropriate selection pressure, genes encoding desired methyltransferase could be selected from a 10^7^‐fold excess of genes encoding another enzyme 29. The aqueous droplets in this case were formed simply by adding the transcription/translation reaction mixture into stirred mineral oil containing surfactant, and were measured to have an average diameter of 2.6 µm. Generating droplets of small volume was important because the translation of methyltransferase could only occur at a sufficiently high local concentrations of gene(s) 29. Despite the large capacity of IVC (>10^10^ compartments available in 1 mL of emulsion), high‐throughput screening and sorting of the created compartments is not possible, due to the incompatibility of the continuous oil phase with FACS. Bernath et al. later proposed further re‐emulsification of w/o emulsions in an aqueous phase to yield water‐in‐oil‐in‐water (w/o/w) double emulsions. This effectively provides the droplets with an external aqueous phase suitable for flow cytometry applications 31. By combining the advantages of miniaturization and FACS, the use of IVC in double emulsions has allowed screening of libraries and hence the optimization of the activity of many enzymes, e.g., beta‐galactosidase 32, cellulase 33, and hydrolase 34; a list that is expected only to grow in the future 26, 35. Furthermore, by creating these man‐made compartments, cell‐based strategies are no longer restricted to enzymes or product that remain trapped inside cells, as previously described 36, 37, 38 (Fig. 3Dii). In recent years, other types of artificial compartments such as the polymer‐based “fur‐shell” compartments 39, 40 and gel‐shell beads (constructed from an agarose core surrounded by a polyelectrolyte shell) 41 have also been established. In contrast to w/o emulsions, these compartments are highly stable in aqueous solutions and therefore can be analyzed by FACS directly. Nevertheless, FACS‐based screening platforms generally still face limitations, with the main challenge being the difficulty in forming and manipulating compartments (e.g., droplet fusion) in a controlled manner. This in turn leads to the formation of polymer aggregates or highly polydisperse emulsions. Since the volume of a droplet has a cubic dependence on its radius, small variations in droplet size can result in large concentration differences between compartments, even though the same amount of product is produced by a compartmentalized enzyme variant. When forming double emulsions, co‐encapsulation of several w/o droplets into one w/o/w droplet can also occur, which further increases the probability of false positives. To overcome these complications, transferring IVC into a chip‐based microfluidic format is highly interesting possibility.

## 3 Droplet‐based microfluidic technologies for DE of enzymes

Droplet‐based microfluidics are flow‐based microfluidic systems that enable the generation and processing of monodisperse w/o droplets at kHz to MHz frequencies 42, with an unprecedented degree of control over droplet properties. A droplet defines a reaction compartment, with the size of each generated droplet being uniformly and precisely defined. This ensures control over the concentration of reagents within droplets, and in turn allows reliable and quantitative analysis of compartmentalized assays. Different protein expression systems, both cell‐based and cell‐free, can be well accommodated when using this technique. Screening of cytoplasmically expressed proteins as cell lysates is also made accessible, simply via the co‐compartmentalization of cells, lysis reagents and substrates. Specifically, advancements within the field over the past 15 years have presented a variety of elegant solutions with respect to handling and manipulating large population of droplets, including the possibility to adjust the chemical or biological payload of already formed droplets, as well as being able to sort them based on different properties 35, 43, 44. One of the most attractive features of droplet‐based microfluidics is its inherent modularity, where unit operations can be designed to represent an individual step within a complex experimental workflow. Accordingly, one or more of these units can be then combined to give integrated platforms tailored to specific biological experimentations, e.g., screening of enzyme libraries, carried out within a single microfluidic device.

### 3.1 Droplet generation

**Figure 4:**
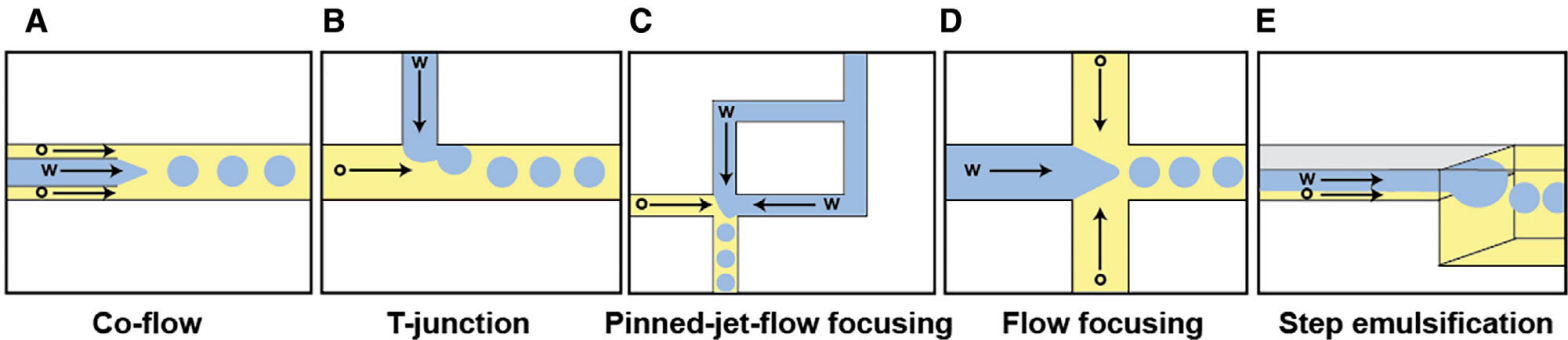
(A)‐(E) Structures of the most commonly used flow‐based droplet generators. Aqueous flow and oil flow are labeled as “w” and “o” respectively, and arrows indicate flow directions. Reprinted and reproduced from 35, with the permission of MDPI Publishing.

To date, a large number of substrate materials have been reported for the fabrication of microfluidic devices/chips, including glass, silicon 45, and a variety of polymers 46, 47, 48, 49, 50. Among all, poly(dimethylsiloxane) (PDMS) 51 has although remained the most widely used material due to its many useful properties, such as being optically transparent, chemically inert and compatible with most biological samples. Moreover, via soft lithography, fabrication of PDMS devices is generally convenient and inexpensive. Using a microfluidic chip, water‐in‐oil (w/o) emulsions can be created, where the aqueous stream is dispersed into an immiscible carrier oil, usually supplemented with a surfactant to stabilize droplets. Microfluidic droplet generators leverage geometrical variations of fluidic structures to produce highly monodisperse (less than 5% of variation in droplet volumes) droplets with volumes on the fL‐nL scale. The most popular geometries are co‐flow structures 52, 53, cross‐flow structures (T‐junctions and pinned‐jet flow focusing 54, 55) and flow‐focusing geometries 56 (Fig. 4A‐D). On chip, the two liquids confined within microfluidic channels are first brought together using hydrodynamic forces (using syringe and pressure pumps), followed by the subsequent formation of droplets at the point of confluence due to shear stress. The choice of droplet generator depends largely on the balance between target droplet volume and production frequency, as monodisperse droplets can only be generated by each geometry within specific flow regimes 57. Among them, the T‐junction is frequently used for the production of larger droplets, whilst the flow‐focusing strategy is preferred for generating droplets of smaller sizes and/or at higher production frequencies. On the other hand, step‐emulsification (Fig. 4E) represents a different type of droplet generation technique, exploiting the sharp change in capillary pressure experienced by two phases when transporting them from a shallower to deeper region of the microfluidic channel to form droplets 58, 59. It is well suited for producing emulsions where viscous liquids are involved 58, as well as the generation of small femtoliter‐sized droplets 60. Apart from the single‐step methods described, two‐step methods have also been adopted for the creation of multiple emulsions (double or triple) in microfluidic devices 61. The simplest multiple emulsions, double emulsions, consist of three phases: the inner phase, the shell phase and the continuous phase. The encapsulated inner phase provides a solvent medium for storing the reagents that enable further droplet processing. The shell phase on the other hand serves as a barrier that physically separates the inner phase from the external environment. The use of chip‐based systems not only provides for control over droplet size, but also the ability to load droplets with multiple reagents at user defined concentrations. For instance, different laminar streams can be combined simply with the use of branched inlet channels prior to droplet production, with the relative concentration of each component being defined by the associated volumetric flow rate ratios. This strategy has been shown to be effective for example in creating droplet barcodes 62, 63 (e.g., by combining different fluorophores) and the study of enzyme kinetics 64, 65, in which co‐encapsulation of multiple reagents yields droplets with unique spectral properties. Upon droplet formation, the mixing of reagents occurs naturally due to the establishment of recirculating streamlines 66, 67, with mixing being enhanced by motivating droplets through winding channels to induce chaotic advection 68, 69. This facilitates direct observation of reaction kinetics with millisecond resolution, thus broadening the range of reactions applicable to on‐chip studies 70.

During droplet generation, the encapsulation of various entities including cells (both mammalian and bacterial), biomolecules such as enzyme or DNA, and beads is yet another significant task in droplet‐based microfluidics. Crucially, and relating to the DE of proteins, the goal is often to encapsulate one bacterial host cell (e.g., *E. coli*) per droplet, such that each of them expresses a distinct enzyme variant of the designed library. In practice, cells are usually loaded together with the aqueous phase, a random process that is passive in nature, yet simple and rapid in operation. This in turn yields a population of droplets with Poissonian‐distributed cell occupancy 71. In this case, to ensure a high percentage of occupied droplets that contain only one cell, diluted samples (λ = 0.1–0.3; such that 9–22% of droplets formed contain only one cell) are preferably used. In spite of the low cell occupancy, most platforms for DE of proteins are developed to rely purely on Poissonian‐based compartmentalization 30. This is because an effective selection throughput of 0.1–1 kHz in a droplet format still surpasses that of traditional MTP systems (∼1 Hz). Besides, within a droplet‐based platform, multiple rounds of screening together with more stringent sorting (selection) thresholds can be implemented to progressively identify the most improved variants.

### 3.2 Unit operations

**Figure 5:**
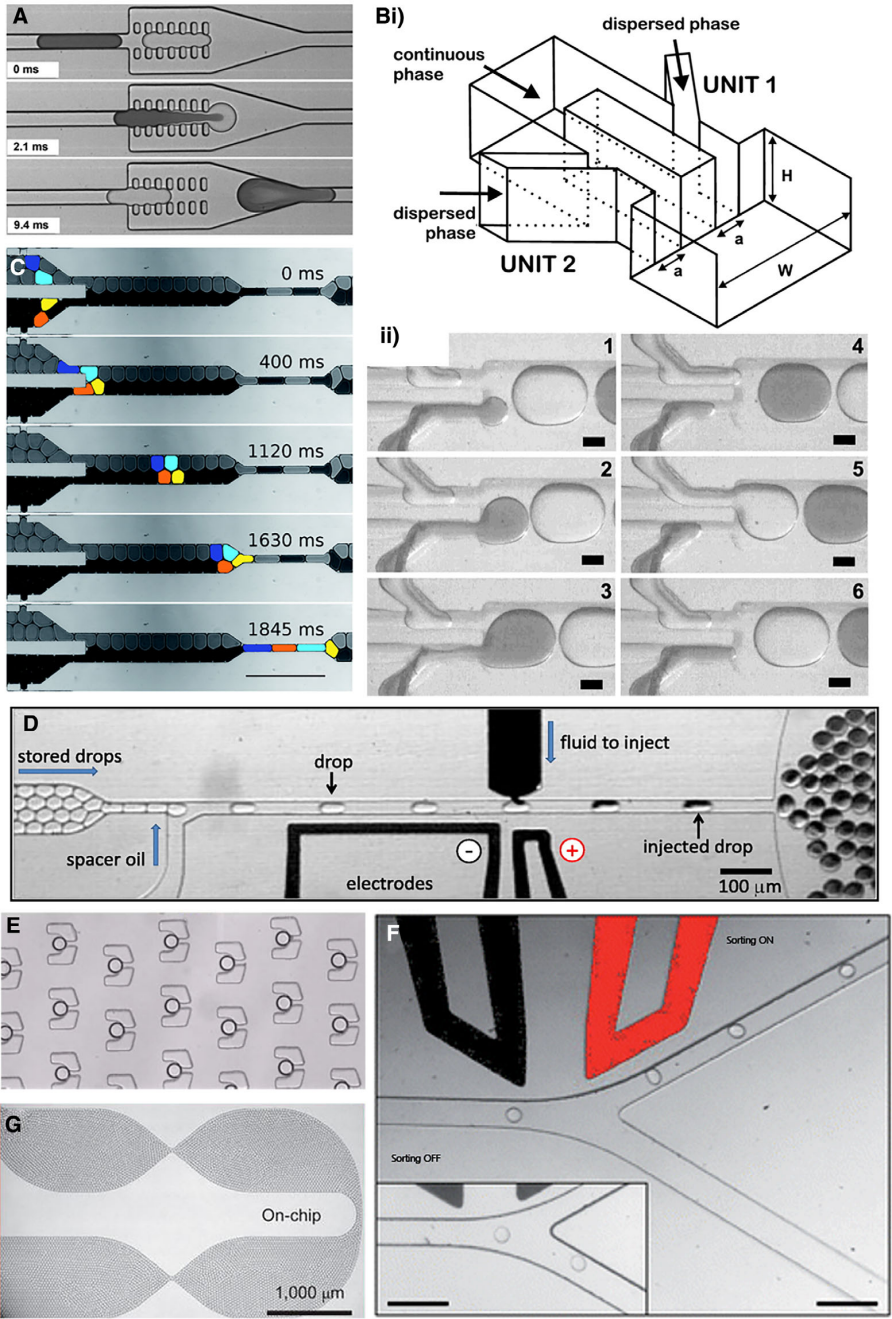
Examples of droplet unit operations. (A) Pillar‐induced droplet merging. Reprinted and reproduced from 72, with the permission of Royal Society of Chemistry Publishing. (B) Self‐synchronising pairwise production of droplets by step emulsification. Reprinted and reproduced from 80, with the permission of American Institute of Physics Publishing. (i) Schematic of microfluidic droplet generator and (ii) time series of optical images displaying formation of two different droplet populations. (C) Reinjection and passive synchronization of droplets resulting in A‐B alternating pattern. Reprinted and reproduced from 83, with the permission of Royal Society of Chemistry Publishing. (D) Electric field‐triggered picoinjection. Reprinted and reproduced from 84, with the permission of National Academy of Sciences of the United States of America Publishing. (E) On‐chip droplet incubation using trap arrays. Reprinted and reproduced from 86, with the permission of Royal Society of Chemistry Publishing. (F) Fluorescence activated droplet sorting (FADS). Reprinted and reproduced from 87, with the permission of Royal Society of Chemistry Publishing. (G) On‐chip long‐term incubation in delay‐lines. Reprinted and reproduced from 88, with the permission of Nature Publishing Group.

Subsequent to their generation, the ability to manipulate droplets in ways mimicking standard analytical procedures is of vital importance. Fortunately, a wide range of functional modules, both passive and active, have been presented for such purposes (Fig. 5).

In order to initiate, modify or even to terminate a compartmentalized reaction, addition of reagents to droplets after formation (equivalent to a pipetting step) is made possible via merging of droplet pairs. Passive methods involve the use of dedicated structures such as pillar arrays 72 or hydrophilic patch inside channels 73, to slow down one droplet before the next approaches so that merging can occur. However, these methods work best when surfactant is not present, which is undesirable when further droplet manipulations are foreseen. Surfactant‐stabilized droplets are naturally more resistant to coalescence. In such situations, merging requires strategies that actively disrupt the droplet interface and cause destabilization, e.g., by applying an electric field 74, 75, 76 or laser pulse 77, 78. Laser‐assisted methods are effective but less frequently used, due to the possibility of damaging biological samples. Importantly, the rate of droplet fusion can reach kHz frequencies 75, 76, thus not significantly restricting the throughput of a complex workflow. To achieve efficient droplet pairing, streams of alternating droplets can be produced using coupled droplet formation schemes 79, 80, 81. Additionally, when handling pre‐formed populations of droplets, reinjection, synchronization architectures can be employed 82, 83. Another elegant solution to modify droplet contents of surfactant‐stabilized droplets is by picoinjection 84, 85. As opposed to merging droplet pairs, fluids can be directly dosed into passing droplets downstream. Injection is activated by applying a controlled electric field to transiently destabilize the droplet/aqueous solution interface, hence allowing the delivery of external solution. The amount of liquid injected is defined by regulating the pressure at the dispensing channel of the injector, as well as the velocity of passing droplets inside the microfluidic channel. Picoinjection can be operated at rates of up to 10 kHz 84, which further increases the potential for high‐throughput biological experimentations.

Once formed, droplets can be incubated in delay lines, for periods from minutes to hours subject to the needs of the assay in hand. For example, enzymatic reactions could range from seconds to hours depending on activity, whilst monitoring cell growth may occur over days. To facilitate incubation at shorter time scales (up to a few seconds), an extended microfluidic channel is simply used, where droplets remain flowing in a single file 68. For longer time scales (from minutes to 1 h), wider and deeper delay lines have been developed, so as to overcome the problem of large pressure drops associated with narrow and lengthy channels 89. If an experiment requires incubation times in excess of 1 hour, droplets can be directly collected and stored in a variety of off‐chip reservoirs such as a Pasteur pipette 87, a syringe 90 or an Eppendorf tube 91. The stored emulsion can then be reinjected on‐chip for further manipulations. Static trapping arrays 71, 86, 92, 93 have also been devised to allow monitoring of single droplets. However, in these situations, the number of traps usually does not exceed several thousand, ultimately limiting throughput.

The ability to perform quantitative analysis and then select specific droplets represents perhaps the most significant operation within a screening platform. An excellent review of analytical methods used in droplet‐based microfluidic systems can be found elsewhere 94. In the current context, only common approaches related to screening of enzyme libraries will be described. Optical detection of laser‐induced fluorescence (LIF) is currently the most‐used approach, not only because of the high sensitivity offered by the technique itself, but also because of the relatively large number of fluorogenic substrates that are commercially available. In addition, detection of multiple colors in parallel can also be achieved, making it possible to register multiple parameters 95. At the same time, LIF is compatible with high‐throughput measurements over short analysis windows (µs‐ms). Most of the reported systems involve the combination of an inverted microscope with a high‐speed camera. The choices of light source include lasers, light‐emitting diodes (LEDs) or mercury lamps, whilst sensitive detection is usually ensured through the use of photomultiplier tubes (PMTs). Indeed, recent studies have also demonstrated the possibility of monitor droplet absorbance 96, a method that is anticipated to broaden the scope of assays compatible for droplet‐based screenings.

Recovery of droplets of interest is carried out on‐chip by active sorting, i.e., by deflecting specific droplets into a dedicated outlet channel on‐chip. To perform sorting, droplets are typically reinjected and spaced by oil stream(s) before passing the point of detection and finally arriving a junction at which the main channel is split into two outlet channels: the “waste” and “collection” channel. Droplets flow per default into the “waste” channel owing to a lower hydraulic resistance. To deflect a chosen droplet, an AC field is applied, which results in a positive dielectrophoretic force on the droplet and hence movement towards the higher electric field region (i.e., ‘collection” channel). Depending on the assay, integration of fluorescence or absorbance detection with dielectrophoresis‐based sorting gives Fluorescence‐activated droplet sorting (FADS) 87 and absorbance‐activated droplet sorting 96 respectively. FADS allows a maximum throughput of 1–2 kHz 87, 97 whilst a sorting rate of 300 Hz can be achieved by absorbance‐activated droplet sorting 96. With an optimised chip design, sorting up to 30000 droplets per second with 99% accuracy has also been reported 98, a throughput that is much more comparable to that of conventional FACS machines (50 kHz). Other reported deflection methods include the application of magnetic fields 99, surface acoustic waves 100, 101, 102 and membrane valves 103, which are detailed in the review by Xi et al. 104. Following sorting, the genes encoding the selected catalysts (or generally droplet contents) can be recovered by breaking the emulsion 105, which can then be fed into further cycles of evolution or ultimately, characterized using conventional techniques. Not surprisingly, microfluidic devices for controlled generation of monodisperse w/o/w double emulsions have also been reported 106, 107, 108. Apart from being compatible with FACS, this technique has great potential with respect to cases where a later release of specific actives within a multi‐component double emulsion droplet becomes necessary, or simply being used as a method to create isolated microreactors 61.

### 3.3 Genotype/phenotype confinement: biocompatibility and droplet stability

The success of any DE experiment is primarily conditioned by the confinement of the genotype‐phenotype linkage. From a microfluidics perspective, long‐term stability of droplets is thus essential; meaning that any uncontrolled coalescence or exchange of components between droplets has to be excluded. Certainly, biocompatibility of all materials and reagents involved, both in prototyping of devices as well as compartmentalization, has to be ensured. Otherwise, cross‐contamination, leakage from droplets or a change in droplet size can obscure assay readout.

Long‐term stability of droplets is almost exclusively facilitated by the use of appropriate surfactants. Surfactants are amphiphilic molecules, bearing groups that have affinity for each phase (aqueous and oil). They are normally supplemented in the continuous phase and upon contact with the discrete phase partition at the interface to enhance stabilization of droplets through the reduction of surface tension. It was proposed that surfactants prevent droplet coalescence mainly by steric hindrance and the establishment of a Marangoni flow, counteracting oil drainage between droplets that are in touch with each other 109, 110. Although many oils and organic solvents can act as carrier fluids, fluorinated oils (e.g., FC40 and HFE‐7500) have been most frequently used in droplet‐based microfluidics, due to biocompatibility requirements and the need to exclude biological impurities. These oils limit droplet exchange, since organic molecules are poorly soluble in them. Furthermore, fluorinated carrier oils are gas‐permeable and compatible with PDMS (PDMS is as well biocompatible and highly gas‐permeable). Owing to the prevalence of fluorinated oils, surfactants used nowadays are chiefly fluorosurfactants consisting of hydrophobic perfluoropolyether (PFPE) tails conjugated with hydrophilic head groups, e.g., polyethylene glycol (PEG) 111. For emulsions with droplet sizes ranging from 10 to 50 µm (as typically generated from a microfluidic device), fluorosurfactants can provide stability for months at room temperature 111. Several of these block co‐polymers are now available commercially, which promotes access to the technology. Nevertheless, droplets should be processed and assayed on the shortest appropriate timescale, in order to avoid undesirable mass transfer that could occur upon prolonged storage. Bulky, charged biomolecules such as DNA are unlikely to pass through droplet interface, whilst smaller molecules such as fluorescent substrates/products are more susceptible to release (into continuous phase) or exchange between droplets over time 112. Leakage is understood to occur most likely via phase partitioning 113 or micelle‐mediated transport, such that free surfactant molecules can form micelles, which consecutively act as cargos to transport molecules from one droplet to another 114. Reducing surfactant concentrations or maintaining droplet‐to‐droplet distance can limit exchange, although this not always applicable, especially when long‐term incubation is required. In fact, reported studies on the exchange kinetics of some fluorescent dyes have shown that retention times could range from days (e.g., fluorescein 112) to seconds to minutes (e.g., rhodamine 112, 115, coumarin 113 and resorufin 114). It has been suggested that increasing hydrophilicity of the dye by introducing more polar groups can reduce micellar transport of molecule 113, 116. Aqueous phases supplemented with additives such as sugars 117 and bovine serum albumin (BSA) 118 have also been found to significantly limit fluorescent dye leakage. Whilst the effect of sugar additives still remains unclear, BSA is believed to act by increasing water solubility and hence reducing diffusion rate of the studied fluorophore 118.

Finally, one must not overlook the importance of controlling channel surface properties. Despite the hydrophobic nature of PDMS, surfaces are usually treated with fluoroalkylsilanes to ensure efficient generation and processing of droplets 119, 120.

## 4 Biology in droplets: current droplet‐based platforms for the DE of enzymes

Over the last two decades, it has become clear that droplet‐based microfluidic platforms (constructed via the integration of functional microfluidic components) enable not only ultra‐high throughput screening of enzyme libraries at low cost, but also effective identification and isolation of novel, improved enzyme variants. Moreover, the breadth of enzyme classes that can be engineered has also been expanded, owing to the rapid increase in the number of chemistries made applicable in droplet formats. Recent examples of library screening campaign performed in microfluidic workflows involve enzyme classes including aldolases 88, 121, hydrolases (e.g., sulfatases, amylases) 41, 122, 123, amino acid dehydrogenase 84, as well as polymerases 124, 125, 126. For more detailed discussions of the relevant reactions, interested readers are kindly asked to refer to review articles drafted by Mair et al. 127 and Bunzel et al. 128.

Nonetheless, it should be mentioned that in some cases, the odds of success are so small that isolation of new enzymes would have been extremely challenging without the throughput gains provided by droplet‐based platforms. For example, Colin et al. reported the application of FADS in the functional screening of metagenomics libraries 123. These libraries consist of environmental DNA from microorganisms, whose biochemistry is otherwise inaccessible in the laboratory environment 129. A library consisting of > 1 million members were screened and 14 new hydrolases for sulfate monoesters and phosphotriesters were identified. These hits could not have been predicted by sequence analysis, as the desired activities have never been ascribed to similar sequences 123. From a screening point of view, the sensitivity of typical agar plate assays would most likely be insufficient to identify hits due to low activities of these enzymes, whilst sophisticated liquid handling systems that afford comparable precision would come at a high price, not to mention with a reduction in overall throughput; thus, leaving FADS to be the most promising method. Evolution of an aldolase described by Obexer et al. represents another application of droplet‐based technologies that has overcome such improbabilities 88. Emerging after six cycles of mutagenesis and screening, the best variant showed a million‐fold improvement in catalytic rate constant (*k*_cat_) compared to the best previously evolved variant 121, resembling rates of natural enzymes. Furthermore, through the choice of appropriate intermediate mutants, experiments in droplets were steered towards a different evolutionary trajectory, identifying enzymes with (*S*)‐enantioselectivity. In contrast, MTP‐based assays have only allowed access to (*R*)‐enantioselective enzymes 130. Starting from the original computational design, a total of up to 10^8^ protein variants have been screened. As emphasized by the authors, such a screen has clearly exceeded the capacity of standard MTP‐based assays (droplets containing fluorescent product can be sorted at frequencies up to 2 kHz, whilst screening 2000 aldolase variants in a conventional plate assay took about two weeks 121), and would not have been plausible without the utilization of FADS.

Directed evolution of proteins using droplet‐based microfluidic technologies has undeniably demonstrated promise. Since most droplet assays still rely largely on fluorescence readouts, the next challenge is perhaps the development of new detection systems to target a wider range of activities, without compromising sensitivity. This could allow protein engineers to move away from fluorogenic substrates, which in many cases show only limited resemblance to the corresponding natural substrates. In light of providing additional readouts, biophysical methods based on surface‐enhanced Raman scattering 131, 132 and droplet morphology are envisioned to be potential candidates. Future commercial availability of droplet sorters would certainly also be a plus, as it would provide research communities that lack resources to microfluidics, the opportunity to implement the technology.

## 5 Conclusion

The commercial enzyme market has grown exponentially in recent years due to improved production technologies, engineered enzyme properties and new application fields. Directed evolution has developed rapidly to become the method of choice for protein engineers to create enzymes having bespoke properties. Advances in the biochemistry of such enzymes, made possible through the development of new technologies, will enhance the knowledge of their structure function properties. For example the development of an efficient screening system for on‐the‐fly screening of novel dehalogenases combined with semi‐rational design will dramatically increase the number of practical applications employing dehalogenases, including biodegradation and biosensing of environmental pollutants, neutralization of warfare agents and industrial biocatalysis 133. In summary, we believe that high‐throughput and high‐sensitivity droplet‐based microfluidics will become the gold‐standard tool for the optimization of computer‐designed enzymes. Indeed, the combination of multidisciplinary approaches with microfluidic technologies will allow the quantitative assessment of evolutionary models providing invaluable insights into how enzymes work, and how they can be designed.

*The authors have declared no conflict of interest*.
